# Pharmacokinetic changes and seizure outcomes in pregnant women taking second-generation antiseizure medications

**DOI:** 10.1186/s42494-026-00275-1

**Published:** 2026-10-27

**Authors:** Nanya Hao, Xiaoli Yi, Renjie Zhao, Xuechen Zhang, Jianxia Dong, Dong Zhou

**Affiliations:** 1 Department of Neurology, West China Hospital, West China Xiamen Hospital, Sichuan University, Xiamem, Fujian China; 2 Department of Clinical Pharmacy, West China Hospital, West China Xiamen Hospital, Sichuan University, Xiamen, Fujian China; 3 West China School of Medicine, Sichuan University, Chengdu, Sichuan 610041 China; 4 West China Hospital of Sichuan University, Chengdu, Sichuan 610041 China

**Keywords:** Antiseizure medication, Therapeutic drug monitoring, Pharmacokinetic changes, Seizure frequency

## Abstract

**Background:**

Effective seizure management in pregnant women with epilepsy poses significant clinical challenges. This study aimed to investigate the pharmacokinetic changes of second-generation antiseizure medications (ASMs) and their association with the deterioration of seizure control in Han Chinese women with epilepsy.

**Methods:**

Patient clinical data including oral daily doses and seizure frequency were acquired from routine clinical practice. Blood concentrations of ASMs determined by therapeutic drug monitoring (TDM) were used to calculate apparent oral clearance (CL) at preconception and each trimester during pregnancy. The ratio of ASM concentration in each trimester to the preconception baseline concentration (RTC) was compared between patients experiencing seizure deterioration and those maintaining stability. Moreover, receiver operating characteristic (ROC) curve analysis was used to identify the threshold RTC value predicting increased seizure frequency.

**Results:**

A total of 52 pregnant patients with epilepsy were initially enrolled in this study. The apparent oral clearance of levetiracetam (LEV) and lamotrigine (LTG) showed a significant increase across all trimesters compared to the nonpregnant baseline. Peak clearance occurred in the third trimester for LTG with a 2.0-fold relative clearance (*P* < 0.05). Similarly, LEV displayed maximum apparent oral clearance increase during the third trimester, reaching 1.6 folds of baseline value (*P* < 0.05). As a result, serum concentrations of LEV and LTG significantly decreased during pregnancy compared to non-pregnant baseline levels (*P* < 0.05). However, the apparent oral clearance and the blood level of oxcarbazepine (OXC) remained stable throughout gestation compared to the preconception baseline. Moreover, the RTC values significantly differed between participants with and without seizure deterioration for all three ASMs, indicating that lower RTCs were associated with seizure worsening.

**Conclusions:**

Our study revealed significant pharmacokinetic fluctuations of LEV and LTG in Chinese pregnant women. This highlights the necessity of early therapeutic drug monitoring for newer ASMs and dose adjustments to maintain blood concentrations near the non-pregnant baseline level in pregnant women with epilepsy.

**Supplementary Information:**

The online version contains supplementary material available at https://doi.org/10.1186/s42494-026-00275-1.

## Background

Epilepsy represents one of the most prevalent neurological disorders, and effective seizure management for pregnant women is facing significant clinical challenges [1, 2]. Epileptic seizures during gestation may lead to severe complications, including blunt trauma, hypoxemia, metabolic acidosis, and spontaneous abortion, which cause a significant threat for both maternal and fetal health [3, 4]. Concurrently, antenatal exposure to antiseizure medication (ASMs) carries teratogenic risks for fetal development [5]. A dose-dependent increase in malformation rates has been observed across most investigated antiepileptic agents, such as carbamazepine (CBZ), lamotrigine (LTG), and valproate [6]. Therefore, therapeutic strategies for pregnant women with epilepsy require a delicate balance between effective maternal seizure control and minimization of fetal ASM exposure.

First-generation ASMs, including phenobarbital, sodium valproate, and carbamazepine, have been associated with significant adverse outcomes such as congenital malformations and neurocognitive impairments in offspring [7, 8]. In contrast, newer-generation ASMs have emerged as alternative therapeutic options for controlling seizures during pregnancy, with improved safety, lower teratogenic risk, and enhanced efficacy in seizure management [9, 10]. Recent clinical evidence has demonstrated that the second-generation ASMs, particularly levetiracetam (LEV), oxcarbazepine (OXC), and LTG, are increasingly prescribed for pregnant women with epilepsy [11].

LEV is mainly eliminated by renal excretion, and approximately one-third of the drug is hydrolyzed in the systemic circulation [11]. After oral administration, OXC undergoes hepatic metabolism to its active metabolite, 10-monohydroxy derivative (MHD), which subsequently crosses the blood-brain barrier to exert antiepileptic effects [12]. LTG is approximately 55% bound to serum proteins and is metabolized primarily by uridine diphosphate glucose glucuronosyltransferases. Maternal physiological alterations during pregnancy, including expanded serum volume, elevated renal blood flow, modified hepatic enzyme activity, and dynamic fluctuations of reproductive hormones (particularly estrogen and progesterone), which collectively alter the pharmacokinetic profiles of ASMs [13, 14].

Studies have reported a nearly three-fold increase in the apparent oral clearance of LTG during pregnancy compared to the pre-pregnancy level [15]. Elevated clearance during pregnancy also results in subtherapeutic serum concentrations of LEV [16]. The blood concentrations of these ASMs decline as pregnancy progresses, with notable individual differences. These pharmacokinetic alterations of ASMs potentially cause two risks: increased seizure recurrence due to subtherapeutic levels and fetal teratogenic exposure to sustained ASM concentrations. Therefore, therapeutic drug monitoring (TDM) is needed to optimize ASM dosing regimens throughout gestation. Previous work recommended monitoring ASM concentrations twice before pregnancy and monthly during pregnancy.

It has been reported that epileptic pregnancies with an over 35% reduction in serum concentrations of LEV and LTG may have a significantly elevated risks of seizure deterioration [17]. Some studies suggest increasing the dose to maintain optimal therapeutic efficacy when the serum concentrations of ASMs deviate by more than 25% from the established therapeutic reference range [18–20].

Due to the differences in genes encoding drug-metabolizing enzymes and transporters among populations of different ethnicities [21], the generalizability of these findings to non-Caucasian populations remains uncertain. Compared to the extensive cohort data in Europe and the United States, evidence from Asian populations was predominantly derived from smaller, single-center studies or retrospective analyses [22, 23]. Prospective, multi-center investigations of the pharmacokinetic curves of ASMs throughout pregnancy remain limited in Han Chinese women. In this study, we investigated pharmacokinetic changes of LEV, OXC, and LTG in each trimester in a Han Chinese cohort. The aim of this study was to quantify alterations in the clearance and serum concentrations of ASMs, evaluate their association with seizure deterioration, and identify preliminary RTC thresholds predicting increased seizure frequency. Our results will provide insights into ethnicity-specific therapeutic drug monitoring strategies and dose adjustment guidelines.

## Methods

### Study population

This study combined both prospective and retrospective designs. Pregnant women taking newer ASMs were enrolled from West China Hospital of Sichuan University. All patients met the following inclusion criteria: (1) age ≥ 16 years; (2) diagnosis of epilepsy according to International League Against Epilepsy in 2017 [24]; (3) gestational age less than 14 weeks at enrollment, and (4) receiving monotherapy or non-interacting polytherapy with LEV, OXC or LTG. Exclusion criteria included: (1) concurrent cardiopulmonary, renal or hepatic dysfunction; (2) serious thyroid disorders, anemia, progressive neurological diseases or taking other medications known to affect the pregnancy outcomes [25]. Patients with poor medication compliance or inability to provide detailed information for ASM doses and seizure frequency were excluded from the study. Patients unable to provide blood concentration data for ASMs were also excluded. This study was approved by the Ethics Committee of Sichuan University (Registration number: 2019[210]) and all participants provided written informed consent prior to enrollment. All participants were informed that their participation was voluntary and that they could withdraw at any time without affecting their clinical care.

### Study design and data collection

**Fig. 1:**
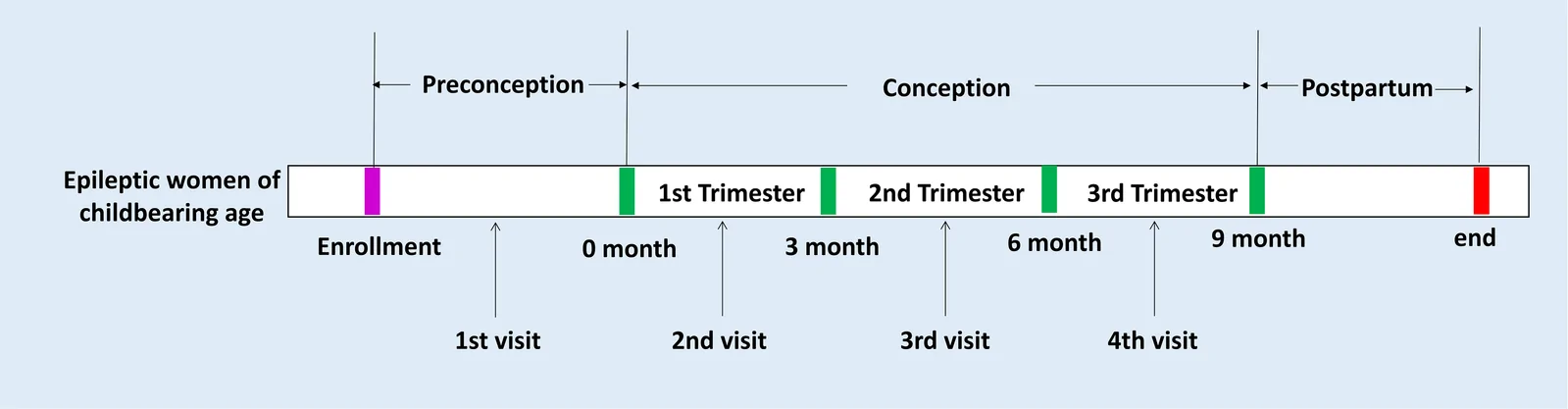
Study design of this study. Participants underwent a total of 4 visits, one during the preconception period, followed by trimestral evaluations at first trimester (conception–3 months), second trimester (3–6 months) and third trimester (6–9 months), respectively. Visits were conducted more frequently when participants exhibited increased seizure activity

The enrolled participants underwent scheduled clinical visits at preconception, first trimester (conception–3 months), second trimester (3–6 months) and third trimester (6–9 months), respectively (Fig. 1). At each visit, peripheral venous blood samples were collected prior to the morning medication dose to measure trough concentrations. Then, serum concentrations of LEV, OXC and its main metabolite MHD, and LTG were determined by Liquid Chromatography-Mass Spectrometry (LC-MS, AB SCIEX Triple Quad™ 4500MD) in Hangzhou Duan Medical Laboratory. The concentrations of MHD were reported together with OXC concentrations [26]. The frequency of therapeutic drug monitoring was increased when seizures worsened, and ASM regimens were adjusted based on the blood levels of ASMs, seizure frequency, and maternal adverse effects. Clinical data, including the daily oral dose, drug administration timing, drug leakage, and seizure frequency, were extracted from the patients’ medical records.

### Alterations of ASM clearance during pregnancy

Blood samples were collected from participants at each clinical visit and absolute serum concentrations of ASMs were measured. Total daily dose and maternal weight were acquired from the medical records. Apparent oral clearance (CL) was calculated as follows: CL = daily dose (mg) /[24 (h) × serum concentration (mg/L)] [4]. Non-pregnant baseline values were derived from measurements obtained before conception or postpartum. A linear mixed-effects model was used to compare CL between baseline value and each trimester of pregnancy [27].

### Alterations of seizure frequency during pregnancy

The seizure frequencies of each participant were prospectively recorded throughout gestational trimesters. Non-pregnant baseline seizure frequency was derived from medical records during a three-month preconception period. To evaluate potential epileptic deterioration, relative frequency changes of epilepsy for each trimester were recorded using a binary classification system: 1, exceeding preconception baseline; 0, equal to or below baseline [28]. Based on the reported correlations between ASM blood levels and seizure control, we calculated ratios of ASM concentrations in each trimester to the preconception baseline concentrations (RTCs) for each participant as follows: RTC = trimester concentration of the drug /preconception baseline [29]. Then, the RTC values were compared between participants with and without seizure exacerbation to examine the correlation between ASM serum concentrations and seizure frequencies. Furthermore, we identified potential threshold RTC values predictive of seizure exacerbation for each ASM by receiver operating characteristic (ROC) curve analysis [30]. The sample size was calculated to achieve 80% statistical power based on established methodological approaches, with adjustment for a 20% anticipated attrition rate.

### Statistical analysis

Data were analyzed with the SPSS Statistics version 19.0 (SPSS, Chicago, IL). Longitudinal changes in ASM clearance rates and serum concentrations were analyzed using a linear mixed-effects model. Two-sample *t*-tests were used to compare RTC values between patients who experienced seizure deterioration and those who did not. No adjustments for multiple comparisons were made. A *P* < 0.05 was considered statistically significant.

## Results

### Clinical characteristics of participants

**Fig. 2:**
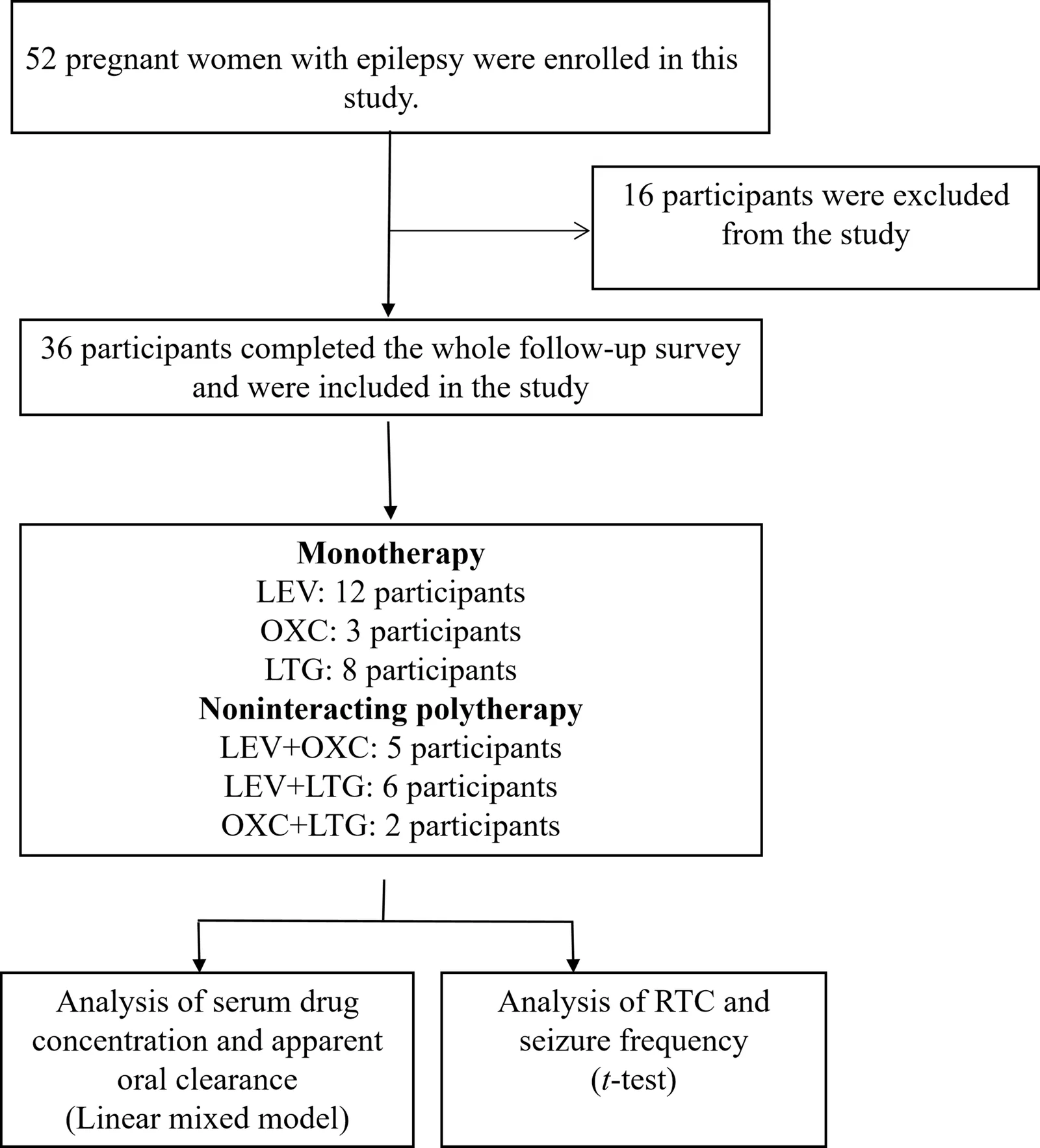
Flowchart of participant recruitment and data evaluation procedures. ANOVA, Single-factor analysis of variance; LEV, levetiracetam; LTG, lamotrigine; OXC, oxcarbazepine

A total of 52 pregnant women with epilepsy were initially enrolled in this study (Table 1; Fig. 2). However, 16 participants were excluded due to poor medication compliance or incomplete blood concentration data. Finally, 36 participants were included (mean age, 28.1 ± 4.1 years, range 22–38; mean body weight, 51.3 kg, range 44–61). Among them, 23 received monotherapy (LEV, OXC or LTG) and 13 were treated with non-interacting polytherapy (LEV + LTG, LEV + OXC or LEV + CBZ). A total of 196 blood samples were collected from these participants to monitor serum concentrations of ASMs, including LEV in 92 samples from 23 pregnancies, OXC in 30 samples from 10 pregnancies, and LTG in 64 samples from 16 pregnancies. In addition, 55.6% (*n* = 20) of the pregnant women had focal epilepsy and 44.4% (*n* = 16) had generalized epilepsy. During gestation, 44.4% (*n* = 16) of the participants experienced seizure deterioration.

**Table 1:** Clinical characteristics of participants in this study

Clinical characteristics	Total number of participants	LEV	OXC	LTG	LEV + OXC	LEV + LTG	OXC + LTG
*n*	36	12	3	8	5	6	2
Average number of seizures during 3 months before conception	\	4	6	3	4	5	7
Seizure type *n* (% of total participants)							
FE	20 (55.6)	7 (58.3)	3 (100)	1 (12.5)	5 (100)	2 (33.3)	2 (100)
GE	16 (44.4)	5 (41.6)	0 (0)	7 (87.5)	0 (0)	4 (66.7)	0 (0)
Seizure worsening *n* (% of total participants)	16 (44.4)	8 (75.0)	1 (33.3)	2 (25)	2 (40)	4 (66.7)	0 (0)
Age, years, mean (range)	28.1 (22–38)	29.3 (23–38)	29.3 (25–33)	28.4 (22–33)	27.6 (20–36)	25.5 (23–31)	28 (27–29)
Body weight, kg, mean (range)	51.3 (46–61)	50.1 (46–55)	54.7 (53–55)	49.9 (45–56)	54.8 (48–61)	50.3 (46–57)	53.5 (53–54)

Abbreviations: LEV, levetiracetam; OXC, oxcarbazepine; LTG, lamotrigine; FE, focal originated epilepsy; GE, generalized originated epilepsy. All parameters refer to pre-pregnancy (baseline) values

### Changes of doses and serum concentrations of ASMs during pregnancy

**Fig. 3:**
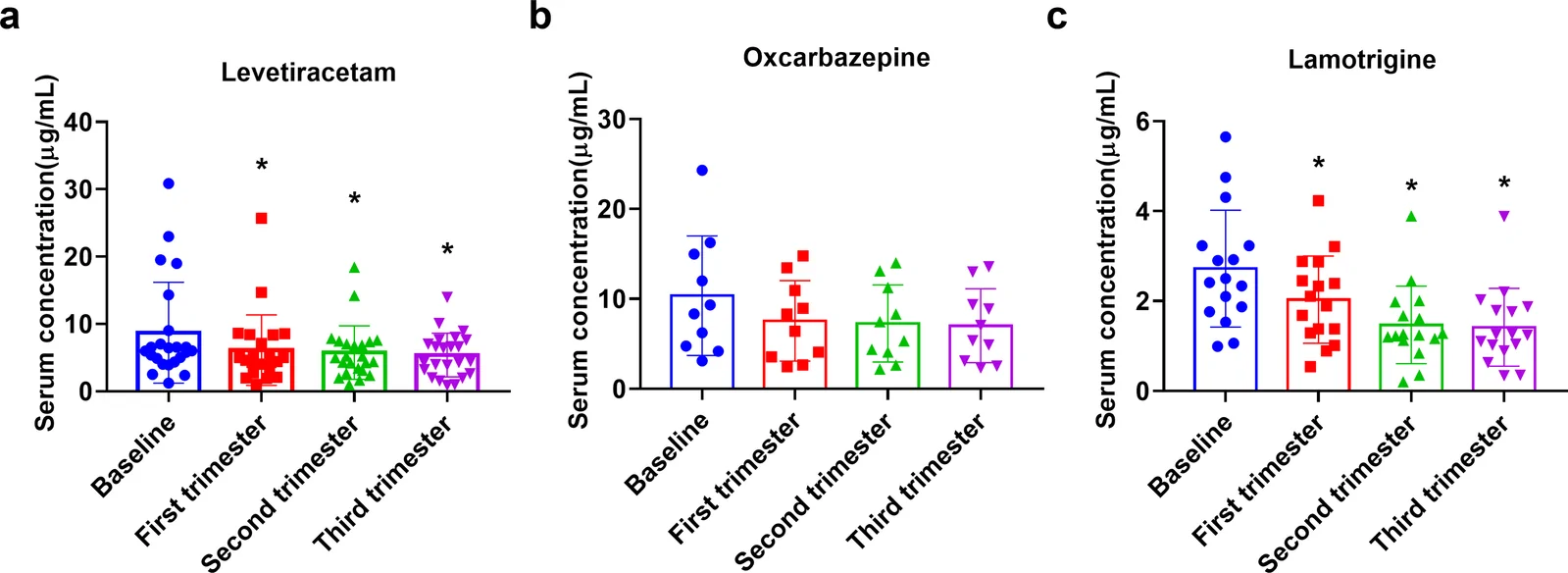
Serum concentrations of ASMs during pregnancy. Data are presented as mean ± SD. *P < 0.05 compared to non-pregnant baseline levels

The total daily dosages and serum drug concentrations of ASMs in the gestational trimesters are presented in Table 2. The mean daily doses of LEV, OXC and LTG were 951.1 ± 384.7 mg (range: 125–1500), 731.3 ± 272.1 mg (range: 300–1500) and 134.4 ± 67.3 mg (range: 25–250), respectively. Compared with the first trimester, the daily doses of LEV and LTG progressively increased during the second and the third trimesters (Table 2). In contrast, the daily dose of OXC exhibited a non-significant tendency toward decrease across the three trimesters (Table 2). The serum concentrations of LEV and LTG exhibited significant reductions during pregnancy compared to the preconception baseline levels (Fig. 3; Table 3), particularly at the third trimester (38.2% reduction for LEV and 47.8% reduction for LTG relative to the baseline, *P* < 0.05; Fig. 3a, c and S1a, c). In contrast, the serum concentrations of OXC did not significantly change during pregnancy compared to the baseline (Fig. 3b and S1b).

**Table 2:** Daily dosages of ASMs before and during pregnancy

ASMs	Mean daily dose, mg (range)			
	Non–pregnant baseline	1st trimester	2nd trimester	3rd trimester
LEV	907.6 (125–1500)	907.6 (125–1500)	972.8 (125–1500)	1016.3 (125–1500)
OXC	810.0 (300–1500)	705.0 (300–1200)	705.0 (300–1200)	705.0 (300–1200)
LTG	135.9 (25–250)	131.3 (25–250)	132.8 (25–250)	137.5 (25–250)

**Table 3:** Serum concentrations of ASMs before and during pregnancy (µg/mL)

ASMs	Non–pregnant baseline	1st trimester	2nd trimester	3rd trimester
LEV	8.70 (1.21–30.84)	6.10 (1.01–25.67)	5.74(0.97–18.43)	5.38 (0.89–18.96)
OXC	10.35 (3.11–24.31)	7.56 (2.45–14.78)	7.28 (2.18–14.01)	7.01 (2.34–13.58)
LTG	2.72 (0.99–5.65)	2.03 (0.54–4.23)	1.46 (0.20–3.89)	1.41 (0.35–3.88)

Data are presented as mean (range)

**Table 4:** Apparent oral clearance of ASMs before and during pregnancy (L/h/Kg)

	Non-pregnant Baseline	1st trimester	2nd trimester	3rd trimester
LEV	0.12 (0.036–0.25)	0.15 (0.053–0.23)	0.16 (0.073–0.278)	0.19 (0.061–0.39)
OXC	0.075 (0.048–0.18)	0.094 (0.070–0.22)	0.095 (0.060–0.32)	0.098 (0.074–0.40)
LTG	0.042 (0.021–0.074)	0.052 (0.015–0.079)	0.075 (0.017–0.17)	0.086 (0.022–0.20)

Data are presented as mean (range)

**Table 5:** Association between RTCs and seizure frequency changes during pregnancy

		1st trimester		2nd trimester		3rd trimester	
Changes in epileptic frequency		Yes	No	Yes	No	Yes	No
LEV	*n* (% of total participants)	6 (26.1)	17 (73.9)	9 (39.1)	14 (60.9)	11 (47.8)	12 (52.2)
	RTC	0.56 ± 0.12	0.84 ± 0.22	0.65 ± 0.27	0.81 ± 0.16	0.68 ± 0.41	0.77 ± 0.18
	P*	0.002		0.09		0.5	
OXC	*n* (% of total participants)	2 (20)	8 (80)	3 (30)	7 (70)	2 (20)	8 (80)
	RTC	0.47 ± 0.51	0.92 ± 0.10	0.61 ± 0.45	0.90 ± 0.07	0.76 ± 0.28	0.84 ± 0.07
	P*	0.02		0.11		0.7	
LTG	*n* (% of total participants)	3 (18.8)	13 (81.2)	5 (31.2)	11 (68.8)	7 (43.8)	9 (56.2)
	RTC	0.52 ± 0.22	0.82 ± 0.09	0.55 ± 0.24	0.63 ± 0.36	0.52 ± 0.17	0.57 ± 0.32
	P*	0.02		0.6		0.7	

* Two-sample *t*-tests were used to compare RTC values between pregnancies with and without increase of seizure frequency in each trimester. A *P*-value < 0.05 was considered statistically significant. RTC: ratios of ASM concentrations in each trimester to the preconception baseline concentrations

### Changes of ASM clearance during pregnancy

**Fig. 4:**
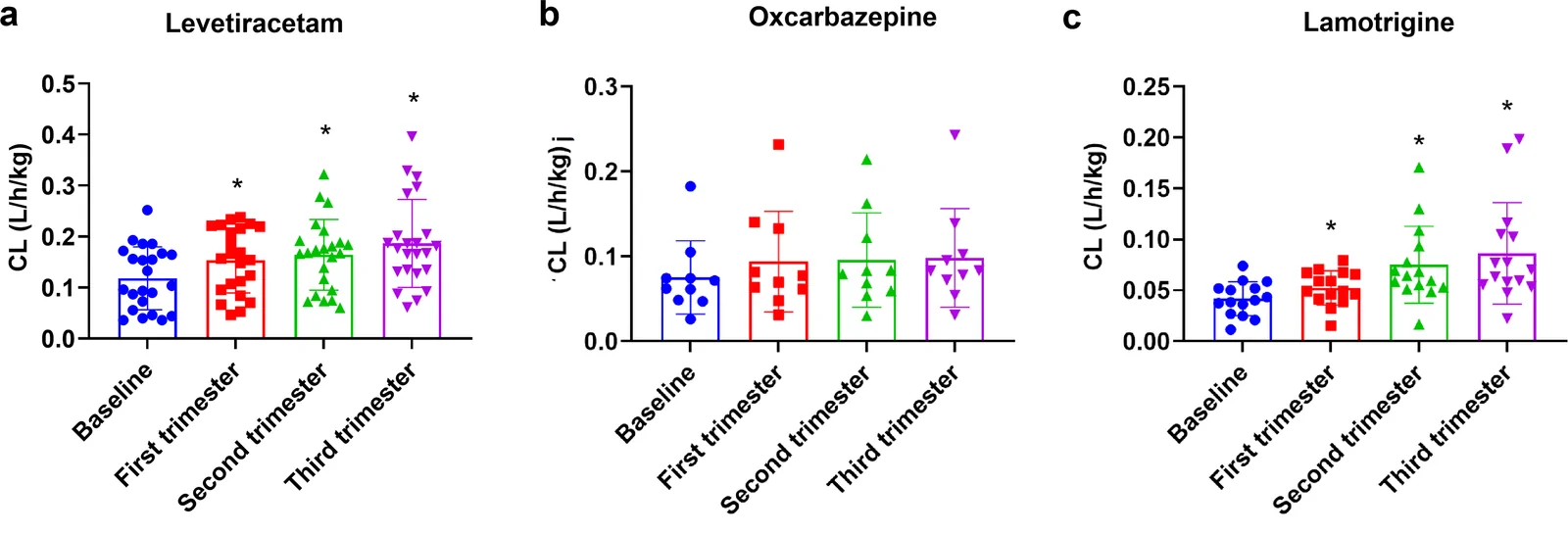
Apparent oral clearance (CL) changes of ASMs during pregnancy. *P < 0.05 compared to baseline. Mean ± SD

As shown in Fig. 4; Table 4, both LEV and LTG showed progressive CL increases throughout gestation. The CL of LEV increased by 30%, 40%, and 60% compared to baseline values in the first, second and third trimesters, respectively (*P* < 0.05, Fig. 4a), despite no significant differences among different gestational stages. LTG displayed the peak CL at the third trimester, reaching 2.0 fold of baseline (*P* < 0.05, Fig. 4c). In contrast, the CL of OXC during pregnancy showed no significant difference compared to the non-pregnant baseline level throughout all gestational stages (Fig. 4B; Table 4)

### Correlations between ASMs serum concentrations and seizure frequency during pregnancy

**Fig. 5:**
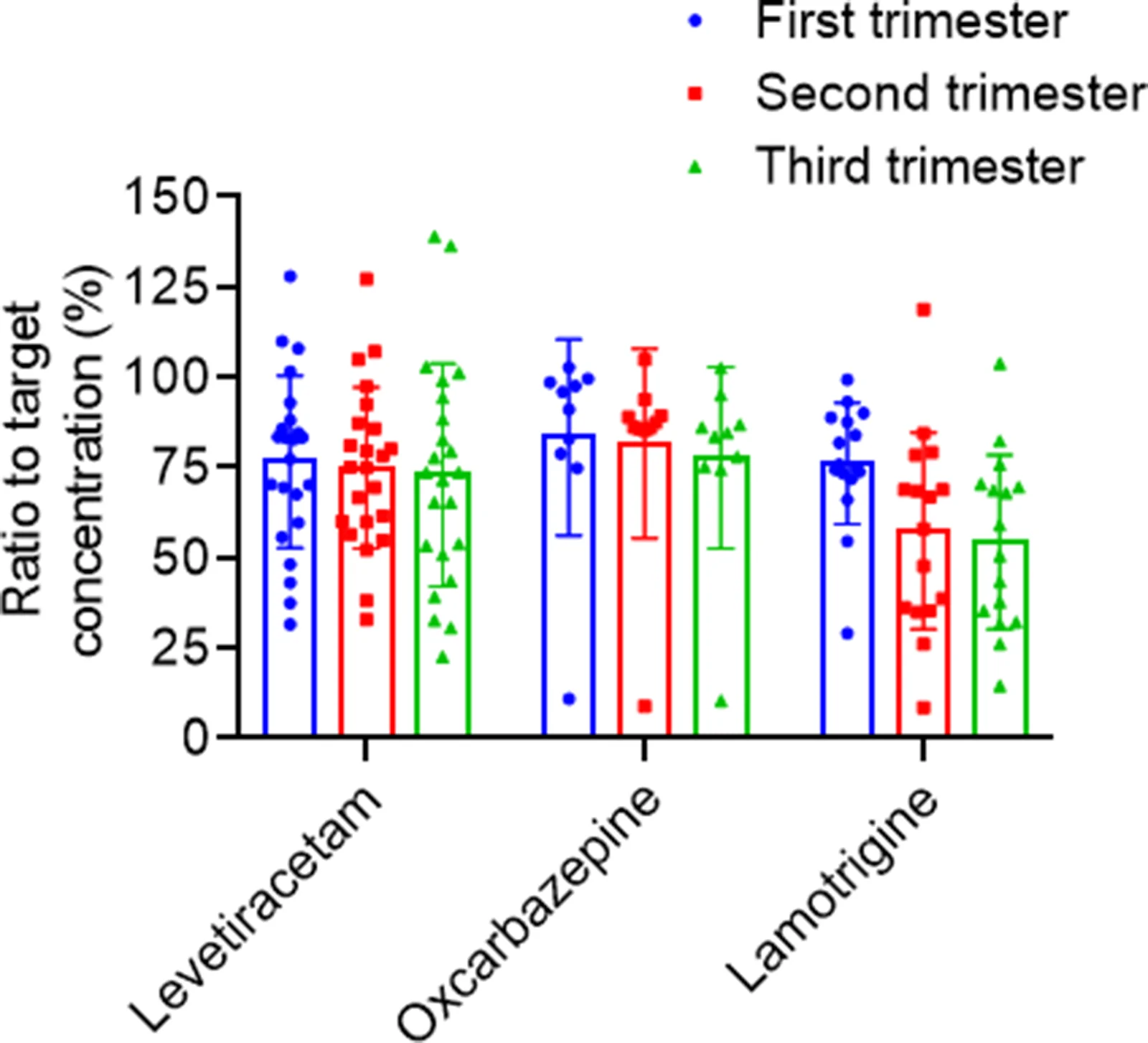
The ratios of ASM concentrations during each trimester of pregnancy to the respective non-pregnant baselines. Mean ± SD

The ratio of serum ASM concentration in each trimester to the non-pregnant baseline concentration was calculated as RTC for each participant. Generally, the RTC values for all three ASMs declined progressively throughout pregnancy (Fig. 5). The RTC values for LEV and OXC were lowest in the third trimester, representing decreases of 27.1% and 22.3% compared to the pre-pregnancy levels, respectively (Fig. 5). Notably, a sharp decline in RTC was observed for LTG during both the second (42.5% decrease) and the third (45.7% decrease) trimesters compared to the baseline (Fig. 5)

**Fig. 6:**
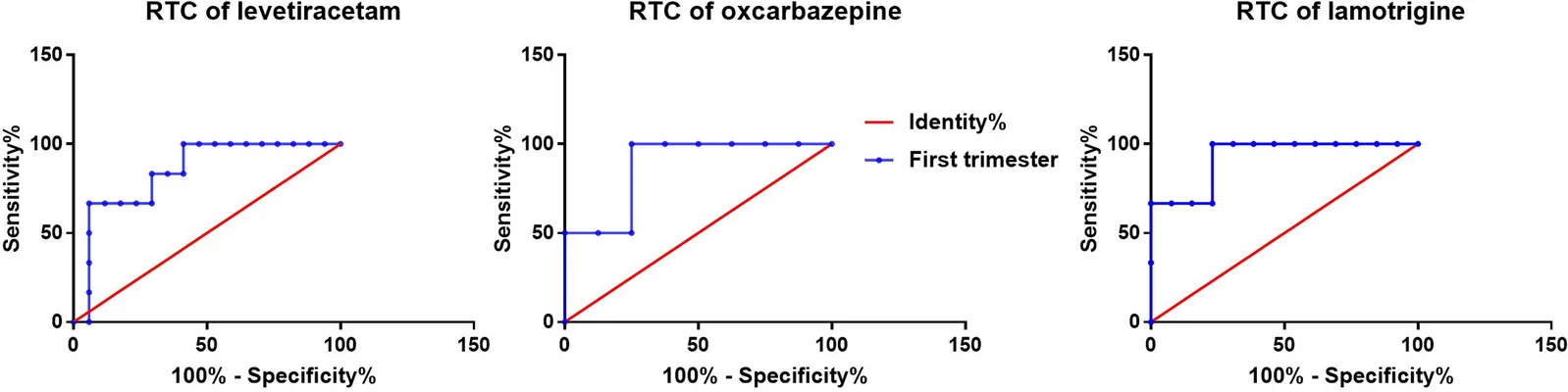
Receiver operating characteristic (ROC) curves were generated to assess RTC thresholds of ASMs for predicting increased seizure frequency. The area under the curve (AUC) values were 0.84 (95% CI: 0.67–1.01, *P* = 0.01) for LEV, 0.88 (95% CI: 0.62–1.12, *P* = 0.12) for OXC, and 0.92 (95% CI: 0.76–1.08, *P* = 0.03) for LTG

To clarify the relationship between seizure frequency and changes of ASM blood level, we compared RTC values in patients with increased seizure frequency during pregnancy versus those without seizure frequency increase in each trimester (Table 5). The results showed that lower RTCs were significantly associated with a higher risk of seizure worsening during the first trimester for all the three ASMs (*P* < 0.05). In contrast, no significant associations were found in the second and the third trimesters. Furthermore, ROC curve analysis revealed RTC thresholds predicting the risk of seizure deterioration (Fig. 6). A > 43% decline in LEV RTC was associated with an increased risk of seizure worsening during the first trimester (Fig. 6). Similarly, serum concentrations below 74% of target levels for LTG and below 85% for OXC were predictive of a higher seizure frequency in the first trimester (Fig. 6)

## Discussion

Previous reports on changes in ASM clearance during pregnancy predominantly involved Caucasian populations. Evidence regarding pharmacokinetic alterations of newer ASMs and dosage adjustments for Asian pregnant women remains limited. Our study provides valuable pharmacokinetic data on second-generation ASMs, oxcarbazepine and lamotrigine, during pregnancy in a Chinese population. Our results confirmed that alterations of ASM pharmacokinetics are common in pregnant women. Using dose- and weight-corrected CL calculated from serum ASM concentration, we found significant increases in oral clearance of ASMs during pregnancy. These increases would result in decreased blood levels of drugs if no compensatory dose adjustments are made, particularly for LTG and LEV monotherapy. We also observed progressive increases in seizure frequency throughout gestation in pregnant patients treated with LEV and LTG.

LEV and LTG are frequently prescribed ASMs for women with epilepsy due to their high oral bioavailability and low teratogenic risk [31, 32]. Our prospective study corroborates prior evidence demonstrating markedly increased CL of LEV and LTG during pregnancy compared to non-pregnant baseline values [33, 34]. We observed elevated CL of LEV as early as the first trimester, with peak CL reaching 1.6-fold of the baseline (*P* < 0.05, Fig. 4A) and a concomitant decrease of serum concentration by 38.2% (*P* < 0.05, Fig. 3A). Since LEV is mainly eliminated by renal excretion in unchanged form [35, 36], the enhanced CL during early pregnancy probably results from increased renal blood flow and glomerular filtration rate [37, 25]. Similarly, LTG exhibited substantial pharmacokinetic alterations: the peak CL reached 2.11-fold of the non-pregnant level (*P* < 0.05, Fig. 4C) and serum concentrations fell below preconception values during the third trimester (Fig. 3C). As LTG undergoes extensive hepatic metabolism via uridine-diphosphate glucuronosyltransferases [38], these pharmacokinetic changes may be partially attributed to estrogen-induced enhancement glucuronidation. In contrast to LEV and LTG, our study detected only minor, non-significant alterations in OXC clearance and serum concentrations during pregnancy (*P* > 0.05; Figs. 3B and 4B). A recent cohort study also reported no statistically significant changes in OXC pharmacokinetics during pregnancy, indicating stable OXC clearance throughout gestation [29].

The relative changes in serum concentrations may be more meaningful than the clearance calculations derived from trough concentrations alone. Our study demonstrated progressive declines in the RTC value of LEV and LTG with advanced gestation. These findings are consistent with previous reports of reduced RTC values for these ASMs during pregnancy [39]. Moreover, our results further confirmed that lower RTC values are associated with seizure exacerbation (Table 5). To clarify the relationship between seizure frequency and ASM concentrations, we compared RTC values per trimester in pregnancies in patients with increased seizure frequency versus those with stable frequency. Our analysis revealed that reductions of LEV serum concentration exceeding 43% predicted worsened seizure control in the first trimester. Similarly, serum concentrations below 74% (LTG) and 85% (OXC) of the non-pregnant baseline concentrations during the first trimester may predict elevated risk of epilepsy exacerbation. These findings underscore the clinical significance of RTC reduction.

Our results indicated that blood concentrations of OXC showed no significant change during pregnancy. This was consistent with previous studies reporting stable OXC levels in pregnant women. However, the extent of fluctuation of OXC blood concentrations, clearance and RTC differed from previous reports. A retrospective study of 135 pregnant women with epilepsy reported 1.9-fold and 2.1-fold increases in clearance compared to baseline value for LTG and LEV, respectively, in Caucasian patients [40]. Similarly, a cohort study of 430 participants revealed 56.1% and 36.8% reductions in dose-normalized concentrations for LTG and LEV during pregnancy in Caucasian women [34]. Similarly, a previous study demonstrated a 31.7% decrease in serum concentration and 46.7% increase in clearance for LEV among Israeli women compared to preconception baselines [29]. Furthermore, the RTC thresholds correlating with seizure worsening in our study differed from established Caucasian thresholds (> 35% decrease). It has been recognized that seizure deterioration during pregnancy may stem not only from declining ASM levels but also from hormonal fluctuations, maternal age, metabolic enzyme polymorphisms, sleep disorders, and stress. Therefore, the discrepancies between our results and prior studies may be attributed to individual variability and ethnic differences.

There are several limitations in this study. First, inconsistent timing of blood collection after the last ASM dose and the lack of monitoring of patient compliance may have influenced the measurements of ASM blood concentrations. Second, the effects of confounding factors, including stress, sleep deprivation, hormonal fluctuations, and pre-conception seizure history, on ASM levels and seizure frequency during pregnancy were not investigated. Additionally, the small sample size constrains the generalizability of our findings. Due to the modest sample size, our analysis of factors associated with seizure exacerbation was restricted to univariate comparisons. Therefore, these univariate findings should be regarded as exploratory and interpreted with caution. Future large-scale, multicenter prospective studies are warranted to validate our findings and the independent predictors of seizure worsening during pregnancy through comprehensive multifactorial analysis. The inclusion of both monotherapy and polytherapy patients may have introduced pharmacokinetic heterogeneity. Larger cohorts are needed to stratify participants by concomitant medication use or restrict analysis to monotherapy for validation. Furthermore, information on potential confounders known to influence seizure threshold during pregnancy, such as medication adherence, sleep deprivation, psychological stress, and hormonal fluctuations, was not collected. The absence of these data precludes causal inference and may introduce confounding bias. Future studies should incorporate prospective collection of these variables using validated instruments to facilitate adjusted analyses.

Nevertheless, our study contributes valuable pharmacokinetic data on ASM variation in a Han Chinese population. Our findings suggest that lower blood concentrations of ASMs correlate with increased seizure frequency, underscoring the necessity of TDM of ASMs starting early in pregnancy. Polymorphisms in genes encoding drug-metabolizing enzymes (e.g., *UGT1A4*, *CYP3A4*, *CYP2C9*) and transporters (e.g., *ABCB1*) vary across populations [41]. The findings from the Han Chinese cohort in this study may not directly apply to other ethnic groups and require external validation before clinical recommendations. Future studies with larger sample sizes are needed to clarify the impact of TDM-guided dose adjustments on seizure control and the risk of epilepsy deterioration during pregnancy.

## Conclusions

Our prospective study revealed a significant increase in CL of LEV and LTG during pregnancy in a Chinese population. Furthermore, lower RTC values of all three ASMs correlate with increased seizure frequency during pregnancy. These results suggest that TDM of ASMs should begin early in pregnancy. Dose adjustments are recommended to maintain blood concentrations near the non-pregnant baseline.

## Supplementary Information

Below is the link to the electronic supplementary material.

Supplementary Material 1

Supplementary Material 2

## Data Availability

All data are available from the corresponding author upon reasonable request.

## Abbreviations

ASMs: Antiseizure medications
CL: Apparent oral clearance
LC-MS: Liquid Chromatography-Mass Spectrometry
LEV: Levetiracetam
LTG: Lamotrigine
MHD: 10-monohydroxy derivative
OXC: Oxcarbazepine
RTC: Ratios of ASM concentrations in each trimester to the preconception baseline concentrations
ROC: Receiver operating characteristic
TDM: Therapeutic drug monitoring

## References

[1] Zhao J, Chen Q, Dong J, Gao M, Ge S, Wang A. Global, regional, and national burden of epilepsy, 1990–2021: a Global Burden of Disease study. J Glob Health. 2026;16:04066. 10.7189/jogh.16.04066 PMC12964325 41789520

[2] Arfman IJ, Wammes-van der Heijden EA, ter Horst PGJ, Lambrechts DA, Wegner I, Touw DJ. Therapeutic drug monitoring of antiepileptic drugs in women with epilepsy before, during, and after pregnancy. Clin Pharmacokinet. 2020;59(4):427–45. 10.1007/s40262-019-00845-2 31912315

[3] Aynacioglu AS, Brockmöller J, Bauer S, Sachse C, Güzelbey P, Ongen Z, et al. Frequency of cytochrome P450 CYP2C9 variants in a Turkish population and functional relevance for phenytoin. Br J Clin Pharmacol. 1999;48(3):409–15. 10.1046/j.1365-2125.1999.00012.x PMC2014334 10510154

[4] Johnson EL, Stowe ZN, Ritchie JC, Newport DJ, Newman ML, Knight B, et al. Carbamazepine clearance and seizure stability during pregnancy. Epilepsy Behav. 2014;33:49–53. 10.1016/j.yebeh.2014.02.011 PMC4040964 24632353

[5] Commission on Classification and Terminology of the International League Against Epilepsy. Proposal for revised clinical and electroencephalographic classification of epileptic seizures. Epilepsia. 1981;22(4):489–501. 10.1111/j.1528-1157.1981.tb06159.x 6790275

[6] Andrade C. Epilepsy, antiepileptic drugs, and adverse pregnancy outcomes, 2: Major congenital malformations with antiepileptic drug monotherapy. J Clin Psychiat. 2024;85(3):24f15432. 10.4088/JCP.24f15432 38959494

[7] Thigpen J, Owens HD. The use of antiepileptic drugs during pregnancy and fetal outcomes. Neonatal Netw. 2022;41(4):226–31. 10.1891/NN-2021-0048 35840331

[8] Veroniki AA, Cogo E, Rios P, Straus SE, Finkelstein Y, Kealey R, et al. Comparative safety of anti-epileptic drugs during pregnancy: a systematic review and network meta-analysis of congenital malformations and prenatal outcomes. BMC Med. 2017;15(1):95. 10.1186/s12916-017-0845-1 PMC5418725 28472982

[9] Usui N, Zhou D, Qin B, Tiamkao S, Cabral-Lim L, Lim KS, et al. Temporal profile of treatment-emergent adverse events in adult asian patients with focal-onset seizures during adjunctive brivaracetam treatment: Post hoc analysis of a phase 3, randomized trial. Adv Ther. 2025;42(11):5639–52. 10.1007/s12325-025-03357-7 PMC12579673 40944847

[10] Fujimoto A, Qin B, Bourikas D, Dickson N, Moseley B, Sano T, et al. Safety, tolerability, and efficacy of adjunctive brivaracetam in japanese and Chinese patients with focal-onset seizures: Interim analysis of a phase 3 open-label extension trial. Adv Ther. 2025;42(9):4335–49. 10.1007/s12325-025-03253-0 PMC12394343 40549270

[11] Fallik N, Trakhtenbroit I, Fahoum F, Goldstein L. Therapeutic drug monitoring in pregnancy: Levetiracetam. Epilepsia. 2024;65(5):1285–93. 10.1111/epi.17925 38400747

[12] Yuan YJ, Zhang S, Yuan YY, Yan XJ, Zhang LL, Ran YW. Pharmacogenomics of oxcarbazepine in the treatment of epilepsy. Pharmacogenomics. 2023;24(6):335–43. 10.2217/pgs-2022-0177 37092337

[13] Kacirova I, Grundmann M, Brozmanova H. Monitoring of lamotrigine concentrations in mothers, colostrum, and breastfed newborns during the early postpartum period. Biomed Pharmacother. 2022;151:113167. 10.1016/j.biopha.2022.113167 35617804

[14] Karanam A, Pennell PB, French JA, Harden CL, Allien S, Lau C, et al. Lamotrigine clearance increases by 5 weeks gestational age: relationship to estradiol concentrations and gestational age. Ann Neurol. 2018;84(4):556–63. 10.1002/ana.25321 30168175

[15] Wang ML, Tao YY, Sun XY, Guo Y, Wang ZY, Cao YF, et al. Estrogen profile- and pharmacogenetics-based lamotrigine dosing regimen optimization: Recommendations for pregnant women with epilepsy. Pharmacol Res. 2021;169:105610. 10.1016/j.phrs.2021.105610 33857625

[16] Westin AA, Reimers A, Helde G, Nakken KO, Brodtkorb E. Serum concentration/dose ratio of levetiracetam before, during and after pregnancy. Seizure. 2008;17(2):192–8. 10.1016/j.seizure.2007.11.027 18180176

[17] Reisinger TL, Newman M, Loring DW, Pennell PB, Meador KJ. Antiepileptic drug clearance and seizure frequency during pregnancy in women with epilepsy. Epilepsy Behav. 2013;29(1):13–8. 10.1016/j.yebeh.2013.06.026 PMC3775962 23911354

[18] Harden CL, Pennell PB, Koppel BS, Hovinga CA, Gidal B, Meador KJ, et al. Management issues for women with epilepsy–focus on pregnancy (an evidence-based review): III. Vitamin K, folic acid, blood levels, and breast-feeding: Report of the Quality Standards Subcommittee and Therapeutics and Technology Assessment Subcommittee of the American Academy of Neurology and the American Epilepsy Society. Epilepsia. 2009;50(5):1247–55. 10.1111/j.1528-1167.2009.02130.x 19507305

[19] Kumaravel J, Sarma P, Suri V, Singh P, Prakash A, Medhi B. Therapeutic drug monitoring of levetiracetam in different trimesters of pregnant women with epilepsy in a tertiary care center: a prospective study. Neurol India. 2024;72(2):358–63. 10.4103/ni.ni_439_22 38691482

[20] Jiménez M, Grau-López L, Ciurans J, García-Esperón C, Fumanal A, Barambio S, et al. Epilepsy and pregnancy. Factors associated with epileptic seizures during pregnancy. Neurologia (Engl Ed). 2023;38(2):106–13. 10.1016/j.nrleng.2020.04.029 36162697

[21] Dorji PW, Tshering G, Na-Bangchang K. CYP2C9, CYP2C19, CYP2D6 and CYP3A5 polymorphisms in South‐East and East Asian populations: A systematic review. J Clin Pharm Ther. 2019;44(4):508–24. 10.1111/jcpt.12835 30980418

[22] Ding Y, Tan XP, Zhang S, Guo Y. Pharmacokinetic changes and therapeutic drug monitoring of lamotrigine during pregnancy. Brain Behav. 2019;9(7):e01315. 10.1002/brb3.1315 PMC6625463 31104352

[23] Duan YF, Yang XM, Zhang MY, Qi XH, Jin Y, Wang ZL, et al. Adaptive dosage strategy of levetiracetam in Chinese epileptic patients: Focus on pregnant women. J Pharm Sci. 2024;113(5):1385–94. 10.1016/j.xphs.2023.12.025 38176454

[24] Specchio N, Wirrell EC, Scheffer IE, Nabbout R, Riney K, Samia P, et al. International league against epilepsy classification and definition of epilepsy syndromes with onset in childhood: position paper by the ILAE task force on nosology and definitions. Epilepsia. 2022;63(6):1398–442. 10.1111/epi.17241 35503717

[25] Thangaratinam S, Marlin N, Newton S, Weckesser A, Bagary M, Greenhill L, et al. AntiEpileptic drug Monitoring in PREgnancy (EMPiRE): a double-blind randomised trial on effectiveness and acceptability of monitoring strategies. Health Technol Assess. 2018;22(23):1–152. 10.3310/hta22230 PMC5960819 29737274

[26] Zhao Q, Jin SY, Liu QY, Zhao ZG, Feng WX, Mei SH. Simultaneous determination of lamotrigine, topiramate, oxcarbazepine, and 10, 11-dihydro-10-hydroxycarbazepine in human blood plasma by UHPLC-MS/MS. Curr Anal Chem. 2020;16(8):1010–21.

[27] Bonate PL. Covariate detection in population pharmacokinetics using partially linear mixed effects models. Pharm Res. 2005;22(4):541–9. 10.1007/s11095-005-2492-z 15846461

[28] Voinescu PE, Park S, Chen LQ, Stowe ZN, Newport DJ, Ritchie JC, et al. Antiepileptic drug clearances during pregnancy and clinical implications for women with epilepsy. Neurology. 2018;91(13):e1228–36. 10.1212/WNL.0000000000006240 PMC6161546 30185446

[29] Tan XP, Li GF, Zhao LM, Guo Y, Liu Y, Yin XT. Pharmacokinetic changes for newer antiepileptic drugs and seizure control during pregnancy. CNS Neurosci Ther. 2022;28(5):658–66. 10.1111/cns.13796 PMC8981429 35037389

[30] Pennell PB, Peng L, Newport DJ, Ritchie JC, Koganti A, Holley DK, et al. Lamotrigine in pregnancy: clearance, therapeutic drug monitoring, and seizure frequency. Neurology. 2008;70(22 Pt 2):2130–6. 10.1212/01.wnl.0000289511.20864.2a PMC3589527 18046009

[31] Mari L, Placidi F, Romigi A, Tombini M, Del Bianco C, Ulivi M, et al. Levetiracetam, lamotrigine and carbamazepine: which monotherapy during pregnancy? Neurol Sci. 2022;43(3):1993–2001. 10.1007/s10072-021-05542-2 PMC8860795 34468899

[32] Stjerna S, Huber-Mollema Y, Tomson T, Perucca E, Battino D, Craig J, et al. Cognitive outcomes after fetal exposure to carbamazepine, lamotrigine, valproate or levetiracetam monotherapy: Data from the EURAP neurocognitive extension protocol. Epilepsy Behav. 2024;159:110024. 10.1016/j.yebeh.2024.110024 39217754

[33] Larcin L, Ngwasiri C, Neven A, Damase-Michel C, Kirakoya‐Samadoulougou F. Real‐world assessment of psychotropic and antiepileptic drug use during pregnancy in belgium: Trends, predictors, and comparative risk of congenital anomalies (2010–2016). Pharmacoepidemiol Drug Saf. 2024;33(10):e70021. 10.1002/pds.70021 39375969

[34] Pennell PB, Karanam A, Meador KJ, Gerard E, Kalayjian L, Penovich P, et al. Antiseizure medication concentrations during pregnancy: Results from the maternal outcomes and neurodevelopmental effects of antiepileptic drugs (MONEAD) study. JAMA Neurol. 2022;79(4):370–9. 10.1001/jamaneurol.2021.5487 PMC8845026 35157004

[35] Chappell K, Kimmons LA, Haller JT, Canada RB, He HJ, Hudson JQ. Levetiracetam pharmacokinetics in critically ill patients undergoing renal replacement therapy. J Crit Care. 2021;61:216–20. 10.1016/j.jcrc.2020.10.032 33217623

[36] Kalaria SN, Armahizer M, McCarthy P, Badjatia N, Gobburu JV, Gopalakrishnan M. A practice-based, clinical pharmacokinetic study to inform levetiracetam dosing in critically Ill patients undergoing continuous venovenous hemofiltration (PADRE-01). Clin Transl Sci. 2020;13(5):950–9. 10.1111/cts.12782 PMC7485952 32223067

[37] Hochbaum M, Kienitz R, Rosenow F, Schulz J, Habermehl L, Langenbruch L, et al. Trends in antiseizure medication prescription patterns among all adults, women, and older adults with epilepsy: A German longitudinal analysis from 2008 to 2020. Epilepsy Behav. 2022;130:108666. 10.1016/j.yebeh.2022.108666 35339390

[38] Wang WY, Battini V, Carnovale C, Noordam R, van Dijk KW, Kragholm KH, et al. A novel approach for pharmacological substantiation of safety signals using plasma concentrations of medication and administrative/healthcare databases: A case study using Danish registries for an FDA warning on lamotrigine. Pharmacol Res. 2023;193:106811. 10.1016/j.phrs.2023.106811 37268178

[39] Schoretsanitis G, Deligiannidis KM, Kasperk N, Schmidt CT, Kittel-Schneider S, Ter Horst P, et al. The impact of pregnancy on the pharmacokinetics of antiseizure medications: A systematic review and meta-analysis of data from 674 pregnancies. Prog Neuro-Psychoph. 2024;133:111030. 10.1016/j.pnpbp.2024.111030 38762161

[40] Chen L, O’brien T, Chen YJ, Abdulaziz A, Lu L, Liu J, et al. Seizure control and pregnancy outcomes in Chinese women with epilepsy: A prospective multicenter cohort study. Epilepsia. 2025;66(5):1573–84. 10.1111/epi.18294 39931893

[41] Biswas M, Jinda P, Sukasem C. Pharmacogenomics in Asians: Differences and similarities with other human populations. Expert Opin Drug Metab Toxicol. 2023;19(1):27–41. 10.1080/17425255.2023.2178895 36755439

