# Supplementary material for: Pharmacokinetic changes and seizure outcomes in pregnant women taking second-generation antiseizure medications
Source: Acta Epileptol. 2026 Oct 27;8:38. doi: 10.1186/s42494-026-00275-1 (PMC13715089; doi:10.1186/s42494-026-00275-1)
Supplement: Supplementary file 1 [file 42494_2026_275_MOESM1_ESM.doc]

**Figure S1**.Serum concentrations of AEDs during pregnancy
